# Updates on the Clinical Evidenced Herb-Warfarin Interactions

**DOI:** 10.1155/2014/957362

**Published:** 2014-03-18

**Authors:** Beikang Ge, Zhen Zhang, Zhong Zuo

**Affiliations:** School of Pharmacy, Faculty of Medicine, The Chinese University of Hong Kong, Shatin, New Territories, Hong Kong

## Abstract

Increasing and inadvertent use of herbs makes herb-drug interactions a focus of research. Concomitant use of warfarin, a highly efficacious oral anticoagulant, and herbs causes major safety concerns due to the narrow therapeutic window of warfarin. This paper presents an update overview of clinical findings regarding herb-warfarin interaction, highlighting clinical outcomes, severity of documented interactions, and quality of clinical evidence. Among thirty-eight herbs, Cannabis, Chamomile, Cranberry, Garlic, Ginkgo, Grapefruit, Lycium, Red clover, and St. John's wort were evaluated to have major severity interaction with warfarin. Herbs were also classified on account of the likelihood of their supporting evidences for interaction. Four herbs were considered as *highly probable* to interact with warfarin (level I), three were estimated as *probable* (level II), and ten and twenty-one were *possible* (level III) and *doubtful* (level IV), respectively. The general mechanism of herb-warfarin interaction almost remains unknown, yet several pharmacokinetic and pharmacodynamic factors were estimated to influence the effectiveness of warfarin. Based on limited literature and information reported, we identified corresponding mechanisms of interactions for a small amount of “interacting herbs.” In summary, herb-warfarin interaction, especially the clinical effects of herbs on warfarin therapy should be further investigated through multicenter studies with larger sample sizes.

## 1. Introduction

Warfarin has been the most commonly used oral anticoagulants ever since its approval in 1954 [1]. Clinically, warfarin is administered as a racemic mixture of the* S*- and* R*-enantiomers.* S*-warfarin is 3–5 times more potent than* R*-enantiomer in anticoagulation effects. Warfarin is highly effective in preventing and treating deep venous thrombosis and can meliorate symptoms in patients suffering from arterial fibrillation, prosthetic heart valves, indwelling central venous catheters, and myocardial infarction [2]. The potency shown in clinical use boosts the studies on the mechanisms of warfarin anticoagulation. The anticoagulation effects are currently believed to be due to warfarin interrupting the vitamin K cycle in liver: in coagulation cascade, activated clotting factors are indispensable for the formation of blood clot. Most of these clotting factors are vitamin K dependent proteins, which suggest that reduced vitamin K is essential for activating clotting factors. Since vitamin K epoxide reductase (VKOR) is responsible for the synthesis of reduced vitamin K, warfarin, by targeting at and inhibiting VKOR, can block the activation of clotting factors and decrease the blood clot [3].

Use of warfarin is still limited despite the strong evidence for its clinical value. This may be accounted by the narrow therapeutic index, warfarin's predisposition to drug and food interaction, and its propensity to cause hemorrhage. Despite the fact that concomitant drug therapy may further increase the risk, complementary and alternative medicines including herbal medicines were widely used in the past decade [4]. Nearly 40% of patients with cardiovascular disease or stroke used concomitant herbs along with their prescribed medications [5]. Herbal medicines and food interaction are now cited as the main cause of adverse events with warfarin. A literature survey over the herb-drug interactions in clinical cases showed that warfarin accounted for 34 of the total 133 cases of interactions, making itself the most frequently involved drug in herb-drug interactions [6]. The incidence of interaction between herbs and warfarin is not yet fully known, and there is no body of reliable information currently available to draw upon when assessing the scale of any possible problem or predicting clinical outcomes. The lack of evidence may be due to under-reporting or unrecognized interactions. In absence of good evidence, speculation has taken its place, and poor quality of available information in turn restricts future study of herb-warfarin interaction.

Herb-warfarin interaction has considerable clinical significance, so it is especially necessary to identify the herbs that interact with warfarin. Unlike what the public usually expect, herbal medicines are not always safe even if they are natural. Adverse events of herbs happen often and are reviewed recently, especially the adverse effects caused by herb-drug interactions [7–9]. Botanical extracts differ greatly from conventional medicines in that the former contain a mixture of many bioactive compounds. The diverse ingredients may result in the prevalence of herb-drug interaction. Knowing how herbs can be used safely and how to reduce the possible risk is the prerequisite for maximizing the benefits derived from herb medicine.

Reports about herb-drug interactions are far from enough, and if exist, they often miss some important items. Several literature surveys showed that interactions of clinical importance were indeed certified by case reports [6, 9–11], but mechanistic study in this field is still limited. A research group assessed the quality of data generated for the study of herb-drug interactions, suggesting that 67% cases were classified as possible interactions, 27% cases were invalid to be evaluated, and only 6% of the cases were well documented [6]. These case reports are insufficient to establish a causal relationship between herb-drug interaction and adverse effects. Patel's group reviewed the published clinical literature from the year 1971 to year 2007, including reported adverse events, descriptions of the clinical case, and case series, to assess the interactions between various herbs and warfarin. Out of 72 documented case reports of herb-warfarin interactions, 84.7% cases were evaluated as possible interactions (61/72) and 15.3% cases (11/72) as probable interactions. Cranberry juice was involved in 34.7% of (25/72) the case reports, as the most commonly involved herb [12]. In addition, western herbs interactions with warfarin are better known, while traditional Chinese medicines (TCM) are rarely studied. According to a literature survey, among 133 cases of herb-drug interactions, St. John's wort was the most common herb studied (37 cases), followed by* ginkgo* and* ginseng* [6]. There were only limited studies on Danshen* (Salvia miltiorrhiza)*, Gegen* (Pueraria lobata)* and several other TCMs. On basis of what have been done on herb-warfarin interactions, the current review aims at complementing the missing points from previous studies as summarized below:

- classification of clinical significance: life threaten, bleeding, INR change, and so forth;
- assessment of evidence reliabilities: highly probable, probable, possible, and doubtful;
- classification of evidence level:* in vitro*, animal, or human;
- summary and classifications of mechanisms for herb-warfarin interactions: pharmacokinetics or pharmacodynamics;
- information related to interactions between TCM and warfarin.

## 2. Methods

In the current review, primary articles released from 1993 and 2013 were searched in both English and Chinese databases including EMBASE, MEDLINE, AMED, Cochrane Systematic Review Database, SciFinder, and CNKI. Search terms were “herb,” “herbal,” “traditional Chinese medicine,” “complementary and alternative medicines,” “warfarin,” “interaction,” “clinical,” and “human study,” Based on the data collected from the search, this paper summarized both traditional Chinese herbs and western herbs involved in clinical herb-warfarin interaction.

### 2.1. Effects of Interaction

A clinical study refers to research using human volunteers (also called participants) who agree to be involved to add to medical knowledge [13]. Outcomes of clinical studies are mainly reported in the form of case reports, case series, clinical trials, and clinical assessments. An overview of literature was conducted in the current study to consolidate clinical evidences on herb-warfarin interactions. Based on these documented clinical evidences, clinical effects of herb-warfarin interactions were classified into either potentiation or inhibition.

### 2.2. Severity of Interaction

Interactions that potentiated or inhibited the effect of warfarin were further rated as major, moderate, minor, or nonclinical (Table 1) according to the ranking scheme developed by Holbrook's group in 2005 [14]. Major potentiation was defined by death, major bleeding, or necessity to stop warfarin therapy entirely. Major bleeding episodes included those that were life-threatening as well as those that led to the loss of at least 2 units of blood in 7 days or less [15]. Moderate potentiation meant that there was an INR change requiring an adjustment in warfarin dosage. In a moderate potentiation, the INR increased to greater than 5.0 or there was an increase in INR by greater than 1.5. Minor potentiation interactions were defined as an INR increase in which no change in warfarin dosage was required. INR increased to less than 5 and the increase of INR was less than 1.5 units in a minor potentiation. Potentiation interactions were classified as nonclinical if the only evidence of warfarin augmentation was a statistically significant increase in warfarin levels without change in INR or clinical status.

Major inhibition interactions were defined by the occurrence of thrombosis. Moderate inhibition (clinically relevant but less than major) indicated a change in INR requiring an adjustment in warfarin dosage. In a moderate inhibition, INR decreased to less than 1.5 or the decrease in INR was greater than 1.5 units. Minor inhibition interactions were defined by an INR decrease requiring no change in warfarin dosage INR decreased to more than 1.5 in a minor inhibition interaction, and the decrease in INR was less than 1.5 units. Inhibition interactions were classified as nonclinical if the only evidence of warfarin inhibition was a statistically significant decrease in warfarin levels. An interaction was defined as having no effect if the interacting drug neither potentiated nor inhibited warfarin's effect in any way described here.

### 2.3. Possibility of Interaction: Reliability of Evidences

Possibility of an interaction was assessed by previously validated criteria and study design that were developed in the first systematic overview on this topic in 1994 [14, 16]. The reliability of evidences was ranked, according to 7 causation criteria, into four levels, with level 1 being highly probable and level 4 being doubtful (Table 2). The lower levels imply that no adequate evidences were available to confirm that interactions will certainly happen.

### 2.4. Mechanism of Herb-Warfarin Interactions

Mechanisms of herb-warfarin interaction are divided into two categories: those involving pharmacokinetics (PK) of warfarin and those involving pharmacodynamics (PD). In addition, we attempted to identify the possible corresponding mechanisms of interactions for relevant herbs.

Pharmacokinetic interactions are those that can affect the process by which warfarin is absorbed, metabolized, and distributed as summarized below. Among these, cytochrome P450s for the metabolism of warfarin is main focus. Other enzymes like UDP-glucuronyltransferase (UGT) may also be involved in herb-warfarin interaction, but little information or literature is available to confirm their effects.

#### 2.4.1. Interference with Warfarin Absorption

Passive diffusion is known as the primary means for warfarin to cross biological membranes. Until now, no transporters have been identified for warfarin absorption in gastrointestinal (GI) environment [17]. Some herbs including Aloe, Jalap, Cascara, and Rhubarb are found to be able to bind with warfarin to affect its absorption [18]. Some herbs may directly cause gastrointestinal membrane erosions leading to risk of hemorrhage after their coadministration with warfarin [19].

#### 2.4.2. Interference with Metabolizing Enzymes of Warfarin

Warfarin is metabolized mainly by cytochrome P450s (CYPs), a very large family of related isoenzymes [20]. Of all those isoenzymes, CYP2C9 accounts for the greatest proportion for metabolism of* S*-warfarin, which is much more potent than *R*-enantiomer clinically. Herbs showing effects on cytochrome P450s, especially on CYP2C9, CYP1A2, CYP3A4, or CYP2C19, will affect the plasma concentration of warfarin, which may be one of the reasons for herb-warfarin interaction [21].

#### 2.4.3. Interference with Protein Binding of Warfarin

Up to 99% of absorbed warfarin is bound to plasma protein, primarily albumin. Herbs competitively bound to albumin will affect the plasma concentration of warfarin, which may also be one of the reasons for herb-warfarin interaction [22]. This, however, is not regarded as the main cause of herb-warfarin interaction [23].

Most of above PK mechanisms for warfarin related interaction were direct interaction such as alteration on CYP enzyme activities and inhibition on protein binding of warfarin. However, there was indeed indirect interaction, for example, when gastrointestinal membrane was damaged.

**Figure 1:**
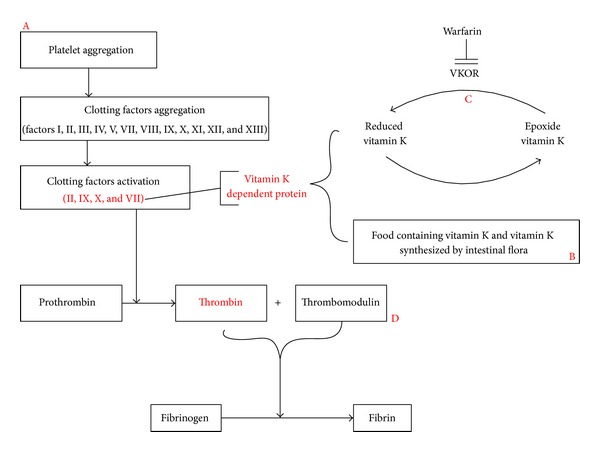
Schematic illustration of the potential pharmacodynamics mechanism for the interaction between warfarin and herbs.

Pharmacodynamic interactions are those where the effects of one drug are changed by the presence of another drug at its site of action. Sometimes the drugs directly compete for particular receptors, specifically VKOR, but often the reaction is more indirect and involves interference with physiological mechanisms. Therefore, pharmacodynamic interactions are more complicated to be classified neatly than pharmacokinetic interactions.  Figure 1 illustrates the process of blood coagulation, pharmacodynamics effects of warfarin, and how herbal components would affect it. It has been shown that pharmacodynamics related mechanisms comprised 79.9% of all the identifiable herb-warfarin interaction mechanisms [5], which are summarized in following four aspects.

#### 2.4.4. Interference with Platelet Function

Platelet aggregation is the first step of coagulation, and a cascade of further platelet activation initiates the formation of blood clot. Reduced platelet aggregation may inhibit thromboxane synthesis [24], thus interfering with clotting mechanisms, decreasing blood coagulation, and prolonging bleeding time [25]. Some antiplatelet herbs, for instance, Ginkgo (particularly ginkgolide B), have been shown to inhibit the binding of platelet activating factor to their receptors on platelet membranes, resulting in reduced platelet aggregation [26, 27]. It can be inferred that the concurrent administration of warfarin with ginkgo may present an additional risk of bleeding.

#### 2.4.5. Altering Gut Vitamin K Synthesis or Containing Vitamin K

*In vivo, *vitamin K can be obtained from two main pathways: being taken from food and being synthesized by vitamin K cycle. Reduced vitamin K is essential for activating several key clotting factors, including II, IX, X, and VII, which are actually vitamin K dependent proteins [28]. Some herbs may stop intestinal flora from synthesizing vitamin K, such as* Thymus vulgaris* and* Allium sativum *[29], thereby enhancing the effect of warfarin. Several herbs such as green tea may contain large amount of vitamin K, which may also result in interaction with warfarin [30].

#### 2.4.6. Interference with Vitamin K Cycle

Besides, synthetized by intestinal flora and taken from food, the primary way for reduced vitamin K synthesis is vitamin K cycle [31]. Some herbs, such as Lapachol [32], affect key enzymes in this circle such as vitamin K epoxide reductase (VKOR), regulating the amount of vitamin K* in vivo*, thereby interacting with warfarin. Warfarin is a synthetic derivative of dicoumarol. Dicoumarol, in turn, is derived from coumarin [33]. Although coumarin itself has no anticoagulant properties, it is transformed into the natural anticoagulant dicoumarol by a number of species of fungi [33]. Therefore, herbs containing coumarin or its derivatives may display similar anticoagulative effects as warfarin. Concurrent administration of warfarin with these herbs may present an additional risk of bleeding.

#### 2.4.7. Interference with Coagulation Cascade

The process of clotting is complex and involves numerous different proteins termed clotting factors (factors I, II, III, IV, V, VII, VIII, IX, X, XI, XII, XIII, protein C, and thrombomodulin) [28, 31]. Some herbs, for instance, Danshen (*Salvia miltiorrhiza*), may affect the expression of thrombomodulin [34], changing blood coagulation* in vivo*, thereby interacting with warfarin.

## 3. Results

Thirty-eight herbs were listed in the current review, including herbs clinically evidenced to interact with warfarin; herbs preclinical evidenced to affect PK or PD of warfarin; herbs containing vitamin K or coumarin; and herbs with similar or opposite pharmacological actions to those of warfarin.

On basis of this overview, effects and severity of each proposed herb-warfarin interaction, as well as the possibility and potential mechanism for those interactions, were abstracted and compiled in Table 3.

### 3.1. Western Herbs

#### 3.1.1. Boldo (*Peumus boldus*)

Boldo was traditionally used for dyspepsia, digestive disturbances, constipation, and rheumatism. Recent research has shown boldine, one of the major active components from Boldo, to be a potent antioxidant [35]. Boldo also contained amount of natural coumarins. But it is unclear whether they have any anticoagulant activity [36]. No relevant pharmacokinetic data of Boldo have been found yet. One case report suggested that it might interact with warfarin. A woman taking warfarin together with 10 drops of Boldo and one capsule of Fenugreek showed a modest rise in her INR from 2 to 3.4. A week after stopping Boldo, her INR had fallen to 2.6 [36]. The mechanism of this interaction remains unknown. Interaction between Boldo and warfarin was defined as* doubtful*.

#### 3.1.2. Cannabis (*Cannabis sativa* L)

Cannabinoids are the major active compounds in Cannabis. Medicinal Cannabis is used to treat chronic conditions, including adjunct and neuropathic pain. There is no experimental evidence for interaction between warfarin and Cannabis [37]. However, a clinical case report described a raised INR and bleeding in a patient who smoked Cannabis (2.5 packs/day for 35 years) while taking warfarin [38].* In vitro* study showed that a major constituent of Cannabis induced CYP2C9 [39]. This would be expected to increase the metabolism of warfarin effects, which is in contrast to the case report. Because of the existence of other factors, it is not reasonable to ascribe the INR change specifically to herb-drug interaction by a single case report. Interaction between Cannabis and warfarin was defined as* possible*.

#### 3.1.3. Chamomile (*Matricaria recutita*)

Chamomile is used for dyspepsia, flatulence, and nasal catarrh [40].* In vitro* study found that the extract of Chamomileinhibited the cytochrome P450 isoenzyme CYP3A4 [41, 42]. However, the effects were weak when compared with the known potent CYP3A4 inhibitor ketoconazole [42]. A study using liver microsomes from rats pretreated with Chamomile for 4 weeks found that CYP1A2 activity was reduced to 39%, when compared with control group [43]. An isolated case of bleeding in a patient taking warfarin with Chamomile products (drinking 4 to 5 cups of Chamomile tea) daily for chest congestion, and using a chamomile-based skin lotion 4 to 5 times daily for foot oedema had been reported [44]. Because of many other factors influencing anticoagulant control, it is unreasonable to identify a drug interaction in a single case report without other supporting evidence. Interaction between Chamomile and warfarin was defined as* possible*.

#### 3.1.4. Chitosan (*Swertia chirayita*)

Chitosan is used as a dietary supplement for obesity and hypercholesterolemia [40]. Chitosan is an absorption enhancer and increased the permeability of hydrophilic drugs across intestinal and mucosal epithelia [45]. One case report suggested that Chitosan might increase the effects of warfarin. In this case, an 83-year-old man stabilized of warfarin treating showed an increased INR from 3.7 to 9, when taking Chitosan 1.2 g twice daily. He was advised to stop this supplement and was subsequently reestablished on warfarin. One month later, the patient restarted the chitosan, which again resulted in a raised INR [46]. Chitosan might impair the absorption of fat soluble vitamins, including vitamin K [46]. Warfarin was a vitamin K antagonist and a reduction in vitamin K would be expected to enhance its effects. Evidence was limited to this case and the mechanism was largely speculative; however, an interaction seemed* possible*.

#### 3.1.5. Coenzyme Q10 (*Theobroma cacao*)

Coenzyme Q10 is usually obtained from parsley, broccoli, peanuts, and grape. Coenzyme Q10 is often taken orally as a supplement to aid treatment of cardiovascular disorders including congestive heart failure, angina, and hypertension [40]. In one controlled study, coenzyme 100 mg daily Q10 for four weeks did not alter the INR or the required dose of warfarin. But another report described decreased warfarin effects in patients taking coenzyme Q10 (30 mg per day for two weeks), with an INR reduction from 2.5 to 1.4 [47]. Similar result was found in another case [48]. A study in rats showed that coenzyme Q10 reduced the anticoagulant effect of warfarin and increased the clearance of both enantiomers of warfarin [49]. The mechanism of interaction between coenzyme Q10 and warfarin was unclear. Coenzyme Q10 may have some vitamin K-like activity [50], which would explain the decrease in INR. Interaction between Coenzyme Q10 and warfarin was defined as* probable*. Until more is known, it is reasonable to increase the frequency of INR monitoring in patients taking warfarin and coenzyme Q10 together.

#### 3.1.6. Cranberry (*Vaccinium macrocarpon*)

Cranberry is commonly used for blood and digestive disorders. Some* in vitro* and animal studies suggested cranberry might affect CYP2C9 and CYP3A4 [51, 52]. However, clinical study found no evidence of significant effects in human [51, 53]. There were some case reports of raised INR and significant bleeding when coadministration of warfarin with Cranberry. One patient died after taking two cups of cranberry juice (approximately 300–400 mL) per day for about six weeks [54]. In the US, a case of major bleeding and a high INR had been reported in man taking warfarin, which occurred shortly after Cranberry juice 710 mL daily was started [55]. In a controlled study, twelve healthy subjects were given Cranberry juice (two capsules three times daily, which is equivalent to 57 g of fruit per day) after warfarin (Coumadin 5 × 5 mg tablets) for fifteen days. INR was increased by 28%, whereas the warfarin pharmacokinetics had no significant difference. The Cranberry juice had no effect on platelet aggregation and pharmacokinetics of either* R-* or* S-*warfarin [56]. The interaction might be therefore via a pharmacodynamics mechanism. For example, the salicylate constituent of commercial Cranberry juice might cause hypoprothrombinaemia [57]. In 2004, on the basis of these case reports, the CSM/MHRA in UK advised patients taking warfarin to avoid drinking juice [58]. They recommended frequently INR monitoring for any patient taking warfarin and having a regular intake of Cranberry juice. Interaction between Cranberry and warfarin was defined as* highly probable*, and no reports with dose-response relationship could be found.

#### 3.1.7. Devil's Claw (*Harpagophytum procumbens*)

Devil's claw is used as bitter tonic and for inflammatory disorders [59].* In vitro*, extract of Devil's claw moderately inhibited the activity of CYP2C8, CYP2C9, CYP2C19, and CYP3A4.* In vitro *study showed that Devil's claw had the greatest effect on CYP2C9 and may increase the effects of warfarin and possible other coumarins [60]. A case report from a 5-year toxicological study described the development of purpura in a patient following the concurrent use of Devil's claw and warfarin (without dosage information) [61]. CYP2C9 was a key enzyme for warfarin metabolism. Limited* in vitro* study suggested that Devil's claw inhibit the metabolism of warfarin, raising its level and potentiating its effect [60]. Clinical evidence of interaction between Devil's claw and warfarin was limited to one case study reporting minor side effects. An interaction seems* possible*, but the evidence is too sparse to make any firm conclusion.

#### 3.1.8. Echinacea (*Echinacea purpurea*)

Echinacea is mainly used in treatment and prevention of common cold, influenza, and other infections.* In vitro* study showed that Echinacea had no significant effect on CYP2C9, CYP1A2, and CYP2D6 [62, 63]. Clinical study showed corresponding result, while a weak inhibition on CYP3A4 was found [64, 65]. In a random study, 12 healthy subjects were given a single dose of warfarin before and after taking Echinacea for 14 days. The AUC of* S*-warfarin decreased by 9%; however, the pharmacokinetic and pharmacodynamics of warfarin had no significant difference [66]. Therefore, Echinacea seemed not to affect warfarin metabolism. Interaction between Echinacea and warfarin was defined as* doubtful*.

#### 3.1.9. Fenugreek (*Trigonella foenum-graecum*)

The seeds of Fenugreek are used mainly for wounds and leg ulcer. It was reported to have hypocholesterol emic and hypoglycemic activity [67]. No relevant data on its pharmacokinetics had been found yet. A case report described that coadministration of one Fenugreek capsule and 10 drops of Boldo increased INR from 2 to 3.4 in patients taking warfarin [36]. However, evidence for this interaction appeared to limit to this one study and it was difficult to identify which of the two herbs is responsible for the increased INR. Therefore, interaction between Fenugreek and warfarin was defined as* doubtful*.

#### 3.1.10. Garlic (*Allium sativum*)

Garlic has been used for respiratory infection and cardiovascular disease. It is believed to have antithrombotic activity [26, 68].* In vitro* studies suggested that Garlic inhibit CYP2C9, CYP3A, and CYP2D6 [69, 70]. Studies in rats suggested Garlic that inhibited CYP2E1 and induced CYP2C9 [40]. However, clinical studies found no significant effects of Garlic on cytochrome P450 isoenzymes [40]. Clinical evidences for Garlic-warfarin interaction were inconsistent with each other. Isolated case reports showed that ingestion of Garlic might cause INR increased apparently and cause bleeding in patients taking warfarin. One patient stabilized on warfarin had a more than doubled INR and showed hematuria 8 weeks after taking Garlic daily. This situation resolved when the Garlic was stopped. Another patient treated with warfarin also showed a more than doubled in INR by taking six Kwai Garlic tablets daily [40]. In contrast, in a placebo-controlled study in 48 patients stabilized on warfarin, there was no change in INR in those receiving 5 mL of aged Garlic extract (Kyolic) twice daily for twelve weeks [71]. Similarly, in a preliminary report of patients taking warfarin, there was no apparent increased risk for bleeding or raised INRs in patients taking Garlic concomitantly [72]. Garlic decreased platelet aggregation, which might therefore increase the risk of bleeding. However, this would not cause an increase in INR, and the mechanism for this effect in the cases reported was unknown [73, 74]. Clinical evidence for Garlic-warfarin information is limited to these reports. Interaction between warfarin and Garlic was defined as* possible*. Serious interactions seem unlikely to happen between warfarin and Garlic. However, it may be prudent to consider a complication of bleeding when Garlic was given with warfarin.

#### 3.1.11. Ginkgo (*Ginkgo biloba*)

The ginkgolides possess antiplatelet and anti-inflammatory properties. It can be used for cerebrovascular and peripheral vascular disorders. The effects of Ginkgo on cytochrome P450 isoenzymes were relatively well studied. It appears that the flavonoid fraction of Ginkgo has more effects on cytochrome P450 isoenzymes than the terpene lactones. And these effects disappear quickly when Ginkgo is stopped [75–77].* In vitro* and rat studies found Ginkgo have effects on CYP2C9, CYP2D6 and CYP2E1. But the effect of Ginkgo on CYP3A4 was unclear and some* in vitro* studies did not appear to be clinically relevant [78–80]. Evidence from pharmacological studies in patients and healthy subjects showed no interaction between Ginkgo and warfarin [81, 82]. However, an intracerebral hemorrhage was reported in an elderly woman when concomitant use of Ginkgo and warfarin in an isolated case. The author of that report speculated that Ginkgo may have contribution to the hemorrhage [83]. There were also a few reports of bleeding [84]. The mechanism of interaction was still uncertain. The interaction between warfarin and ginkgo was* possible*. Evidences are insufficient to justify advising patients taking warfarin to avoid ginkgo, but patients are suggested to monitor their INR when co-administrated ginkgo with warfarin.

#### 3.1.12. Ginger (*Zingiber officinale* Roscoe)

Ginger has anti-inflammatory, antispasmodic, and antiplatelet activities [85]. Pharmacological studies suggested that Ginger does not increase the anticoagulation effects of warfarin [86]. However, a case report described a markedly raised INR in a woman taking warfarin and pieces of Ginger root together (without dosage information) [82]. Moreover, in a prospective, longitudinal study of patients taking warfarin and herbal product, there was a statistically significant increased risk of bleeding events in patients taking warfarin and Ginger [72]. In a randomized, crossover study in twelve healthy subjects, three Ginger capsules (*Blackmores Travel Calm* Ginger capsule containing an extract equivalent to 400 mg of Ginger rhizome powder) taken three times daily for two weeks did not affect either the pharmacodynamics or pharmacokinetics of a single 25 mg dose of warfarin taken on day seven [82]. Ginger was believed to be an herb that interacts with warfarin on the basis of its inhibition on platelet aggregation* in vitro*. However, results of* in vitro* studies cannot be simply extended to clinical [84]. Despite Ginger being cited as an antiplatelet aggregation herb, there was limited evidence suggest that it can increase warfarin anticoagulation effect. There was only one case report showed markedly increased INR for patient coused warfarin and Ginger tea. Without ruling out effects of other factors, it is unreasonable to ascribe this change to Ginger-warfarin interaction. Based on above, interaction between warfarin and Ginger wasdefined as* probable*.

#### 3.1.13. Ginseng

Ginseng is used to enhance the body's resistance to stress and to improve mental and physical performance [40]. The active constituents of Ginseng are mainly ginsenosides that are believed to inhibit the platelet aggregation and thromboxane formation [87]. One study showed that Ginseng (Ginseng capsules three times daily for two weeks) modestly decreased the anticoagulant effects of warfarin (INR decreased from 3.1 to 1.5) [88], and another patient taking warfarin was found to have thrombosis with a subtherapeutic INR of 1.4 (without dosage information) [89]. A clinical study also showed that American Ginseng (1.0 g, twice daily for three weeks) modestly reduced the AUC warfarin in healthy volunteer with INR slightly decreased [90]. In contrast, in a randomized, crossover study in 12 healthy subjects, Ginseng capsules 1 g three times daily for two weeks did not affect either the pharmacokinetics or pharmacodynamics (INR) of a single 25 mg dose of warfarin [91]. The ginsenosides have been reported to inhibit CYP1A2 to some extent, and other ginsenosides metabolites had been found to exert an inhibitory effect on CYP3A4, CYP2D6, or CYP2E1 [40]. Study in rats failed to find any evidence of an interaction between warfarin and Ginseng. Based on the above, interaction between Ginseng and warfarin was defined as* possible*. It was unclear why Ginseng might reduce the efficacy of warfarin.* In vitro* study found that* Panax ginseng *contained antiplatelet components that inhibit platelet aggregation and thromboxane formation [92]. It was reasonable to recommend the caution while combining Ginseng and warfarin.

#### 3.1.14. Grapefruit (*Citrus paradisi*)

Grapefruit juice can inhibit CYP3A4 irreversibly and cause drug interactions in a relatively low dose [93]. A couple, both well stabilized on warfarin, took some drops of Grapefruit seed extract products (*Estratto di Semillas di pompelmo*, Lakshmi, Italy) for 3 days. The women developed a minor hematoma and the man had a raised INR of 5.1 [94]. Mechanism of the interaction was inferred to be that Grapefruit inhibited CYP2C9 and CYP3A4 and therefore affected the metabolism of warfarin [95]. Data presented in these cases, backed by* in vitro* data, supported that Grapefruit had the potential to interact with warfarin, and this interaction wasdefined as* possible*. On this basis, it would probably be prudent to avoid coadministration of warfarin with Grapefruit or for concurrent use to be monitored closely.

#### 3.1.15. Green Tea (*Camellia sinensis*)

*Camellia sinensis* has been reported to contain high amounts of vitamin K, conflicting evidences indicating various amount of vitamin K in green tea [30]. It is true that the dried leaf of* Camellia sinensis* is rich in vitamin K, containing as much as 1428 *μ*g/100 g of leaves. However, brewed tea only contains about 0.03 *μ*g of vitamin K per 100 g of brewed tea [96]. A 44-year-old man taking warfarin 7.5 mg once daily for stroke prevention had a significant decreased INR from 3.79 to 1.37, which was attributed to the ingestion of green tea (0.5–1 gallon). On discontinuation of the green tea, the patient's INR increased to 2.55. This interaction might be attributed to the vitamin K contained in the tea [97]. In another case, a 67-year-old white female was prescribed warfarin at a dosage of 32 mg/week atrial flutter. After receiving warfarin for 3 months, the patient stopped drinking black tea (dose not known). Within one week after discontinuing the black tea, the patient's INR of increased from 1.7 to 5.0. The weekly dose of warfarin was subsequently decreased to 26 mg/week [98]. The antagonism of warfarin by green tea has been reported to be mainly attributed to the vitamin K contained in the tea. However, there is evidence which shows the antiplatelet effect of green tea. The compounds, including catechin and caffeine, in green tea may stop arachidonic acid release from platelet and thereby inhibit blood clot formation [99]. The reason for this interaction is still unclear, but patients are suggested to take this* possible* interaction into attention when receiving warfarin treatment.

#### 3.1.16. Melilot (*Melilotus officinalis*)

Melilot is used mainly to treat inflammation, oedema, and capillary fragility [100]. There was no relevant pharmacokinetic data for Melilot. In one case, a 66-year-old women's INR rises from 2 to 5.8 after 7-day intake of Melilot with acenocoumarol [101]. Another report showed a woman developed a prolonged prothrombin time when taking large quantities of a herbal tea containing Melilot [102]. However, experimental evidence for this interaction was blank. The mechanism of interaction was unclear. Some studies inferred that the natural coumarins contained in Melilot might be a reason for the interaction between warfarin and Melilot. On the basis of limited case reports and lacking of mechanistic study, this interaction may only be considered as* doubtful*.

#### 3.1.17. Parsley (*Petroselinum crispum*)

Parsley is used as a diuretic and may significantly ameliorate symptoms of arthritis, rheumatism, and inflammatory disorders. A study showed that Parsley reduced the content of cytochrome P450 in rat liver [103]. There were no experimental data of the interaction between Parsley and warfarin. Only one case report showed that a 72-year-old man taking warfarin had a raised INR of 4.43, and after that he stopped 7-year intake of herbal products containing Parsley [104]. It was therefore likely that the Parsley contained sufficient vitamin K to antagonize the effect of warfarin. Interaction between warfarin and Parsley can be considered as* doubtful*. Nevertheless, some consider that increased INR monitoring is required in any patient wanting to start any herbal medicine or nutritional plant.

#### 3.1.18. Pumpkin (*Cucurbita pepo*)

Pumpkin is traditionally used to treat tapeworm infection and has been recently used to treat benign prostatic hyperplasia [105]. There was no experimental data of interaction between Pumpkin and warfarin. One clinical case report showed that an elderly man stable taking warfarin who had a raised INR to 3.4 after starting herbal products containing Pumpkin for 6 days [106]. The mechanism was unclear but may be due to the amount of vitamin K contained in Pumpkin. Because of the limitation of information, the interaction between warfarin and Pumpkin might be considered as* doubtful*.

#### 3.1.19. Red Clover (*Trifolium pratense*)

Red clover is used to reduce the symptoms of the menopause. It could also be used for mastalgia, premenstrual syndrome, and cancer prevention. An* in vitro* study showed that Red clover reduced the activity of CYP1A2, CYP2C8, CYP2C9, CYP2D6, CYP2C19, and CYP3A4 and especially inhibited CYP2C8 and CYP2C9 [60]. Some reviews listed Red clover as having the potential to increase the risk of bleeding or potentiate the effects of warfarin, based on the fact that Red clover inhibited CYP2C9 and contained natural coumarins [107]. But there was only one case report support this statement A 53-year-old woman developed spontaneous subarachnoid hemorrhage when she was taking warfarin with herbal supplements containing Red clover, Chinese Angelica, Ginseng, and others for 4 months [108]. With this case reports, it is not possible to identify which, if any, of these constituents in the herbal supplements might have contribution to the hemorrhage. Therefore, the interaction between Red clover and warfarin was defined as* doubtful*.

#### 3.1.20. Saw Palmetto (*Serenoa repens*)

The primary use of Saw palmetto fruit is to treat benign prostatic hyperplasia. It can also be used as an endocrine agent.* In vitro *studies suggested that Saw palmetto inhibits some cytochrome P450 isoenzymes, including CYP2D6, CYP2C9, and CYP3A4 [109]. However, clinical studies found that Saw palmetto did not appear to have a clinically relevant effect on the majority of cytochrome P450 isoenzymes [110]. A case report showed that the INR of a 61-year-old man modestly increased from 2.4 to 3.4, and after that he took Saw palmetto containing product (Curbicin, five tablets daily for six days) together with warfarin [106]. In addition, Saw palmetto (without dosage information) has been reported to cause excessive bleeding in a 53-year-old man undergoing a surgical procedure to remove a brain tumor [111]. Experimental evidence found that Saw palmetto inhibited the CYP2C9, which may be one of the reasons for interaction between warfarin and Saw palmetto [109]. However, evidences were limited to case reports and an experimental study of unknown clinical relevance, which reduced the possibility of interaction. Therefore, the interaction between Saw palmetto and warfarin was defined as* doubtful*.

#### 3.1.21. Soya (*Glycine max* Merr.)

Soya is widely used in Japanese and Chinese cuisine. There are numerous purported benefits of Soya protein, including hyperlipidemia, menopausal symptoms, and osteoporosis [112]. An* in vitro* study showed that Soya bean products inhibited CYP3A4 and CYP2C9 [113, 114]. However, the findings of* in vitro* studies cannot be directly extrapolated to clinical situations. Fermented Soya bean products contain high level of vitamin K and may therefore decrease the activity of warfarin and related anticoagulants [115]. Experiments in rabbits found that Natto, a Japanese food made from fermented Soya bean containing high levels of vitamin K, strongly antagonized the effects of warfarin [116]. Clinical case reports also showed marked reduction in effects of acenocoumarol, a warfarin derivative anticoagulant, when coadministrated with Natto (100 g/day) for two weeks [117]. In this study no dose-response relationship could be concluded. In another clinical study, Soybean protein also modestly reduced the effects of warfarin [116]. A similar interaction was reported for a 70-year-old man whose INR decreased from 2.5 to 1.6 when taking 480 mL Soy milk daily after warfarin treatment for four weeks [115]. Mechanistic study suggested that the antagonistic interaction between warfarin and Soya was due to the high level of vitamin K in Soya and the inhibition effect of Soya extracts on CYP2C9 and CYP3A4. The interaction between warfarin and fermented Soya bean production was established and marked and was* highly probable* to be clinically relevant in all patients.

#### 3.1.22. St. John's Wort (*Hypericum perforatum*)

St John's wort is an herbal medicine mainly used for treatment of depression. An amount of interactions related to St John's wort have been reported in clinical case reports.* In vitro *studies demonstrated that St John's wort inhibited CYP2C9, CYP2D6, and CYP3A4 [41, 118]. Paradoxically,* in vivo *studies found that St John's wort induced CYP2D6, CYP2E1, and CYP3A4 [119, 120]. There are several case reports suggesting that coadministration of St John's wort decreased the effects of warfarin. From 1998 to 1999 period, the Swedish Medical Products Agency (MPA, Uppsala, Sweden) has received seven case reports of a reduced anticoagulant effect and decreased INR of warfarin associated with coadministration of St John's wort [121]. In a randomized, crossover study in 12 healthy subjects, one tablet of St John's wort (each tablet containing standardized dry extract equivalent to 1 g* Hypericum perforatum* flowering herb top, 0.825 mg hypericin and 12.5 mg hyperforin) three times daily for two weeks modestly decreased the AUC of both* R*- and* S*-warfarin by about 25% after a single 25 mg dose of warfarin [91]. However, in another case, an 85-year-old patient taking warfarin 5 mg daily was reported to develop upper gastrointestinal bleeding one month after starting St John's wort (without dosage information) [122]. Until now, the potential interactions between warfarin and St John's wort have not been systemically investigated. Concomitant intake of St John's wort was associated with a loss of anticoagulant activity in patients stabilized on warfarin. Although no thromboembolic episodes occurred, the decrease in anticoagulant activity was considered clinically significant. Anticoagulant activity was restored when St John's wort was terminated or the warfarin dose was increased. These observations suggest an increased clearance of warfarin, possibly due to the induction of CYPs, particular CY2C9, and 3A4. Based on the above findings, a modest pharmacokinetic interaction between St John's wort and coumarins would be established, which might be clinically important in some patients. The possibility of interactions between St. John's wort and warfarin has been considered as* highly probable*. And it is recommended to closely monitor INR in patients taking warfarin after ingestion of St. John's wort.

### 3.2. TCM Herbs

Among the reported herbs,* Ginseng, Andrographis Paniculata,* and* Melilotus Officinalis* are also commonly used as TCM. In addition, we summarized a few more TCM herbs commonly used in Chinese population that may have interactions with warfarin as follows.

#### 3.2.1. Chinese Angelica (*Angelica sinensis*)

Chinese Angelica is mostly used for the treatment of menopausal symptoms, menstrual disorders, hypertension, and allergic conditions [123]. A study in rabbits showed an increase in prothrombin time but no changes in the pharmacokinetic parameters of warfarin [124]. In contrast, most experimental evidences showed that Chinese Angelica inhibited CYP2C9 and CYP3A4, which indicated the potential risk of interaction between Chinese Angelica and a wide range of conventional drugs [125]. In one clinical case, the INR and prothrombin time of a 46-year-old woman doubled after Chinese Angelica (one 565 mg tablet 1-2 times/day for four weeks) and warfarin treatment. And these indexes went back to normal when stopped Chinese Angelica [126]. Another case report also described a very marked increase of INR to 10 when coadministration of warfarin with Chinese Angelica for a month (without dosage information) [127]. The reasons for this interaction are not fully understood. A high level of coumarin derivatives may be included. Other studies suggest that the herb may inhibit CYP2C9, which is the main route of warfarin metabolism. On basis of limited clinical evidence, the interaction between Chinese Angelica and warfarin is not fully established and may be defined as* probable*. More studies are needed to certify this interaction. However, for safety, the use of Chinese angelica should be avoided unless the effects on anticoagulation can be monitored.

#### 3.2.2. Danshen (*Salvia miltiorrhiza*)

Although not commonly used in the western cultures, Danshen (the dried root of* Salvia miltiorrhiza Bunge*) is a very popular TCM recommended in China and many other Asian countries for promoting circulation and improving blood flow. Primary clinical application of Danshen is treatment of various cardiovascular and cerebrovascular diseases, including angina pectoris, hyperlipidemia, and acute ischemic stroke [128]. Previous studies indicated that Danshen extracts could increase the absorption rate, area under the plasma concentration-versus-time curve (AUC), as well as the maximum concentration (*C*_max⁡_) of warfarin, and reduce the elimination half-life (*t*_1/2_) in rats. Danshen injection was reported to significantly increase the plasma concentration of warfarin in rats [129, 130]. Plenty of rat/mouse* in vivo* and cell-based* in vitro* studies showed inconsistent effects of Danshen products on cytochrome P450 isoenzymes. In a study on mice, a commercial pharmaceutical extract of Danshen induced the activity of CYP1A2 by about 60%. A purified extract of tanshinone IIA had a similar effect in this study [131]. In converse, another study using mice and human liver microsomes showed that tanshinone IIA inhibited CYP1A2 [132]. In clinical study, INR of patients taking warfarin significantly increased after ingestion of Danshen. There are several case reports about the warfarin-Danshen interaction. A 62-year-old man stabilized with warfarin had a raised INR to more than 8.4 after consuming Danshen extract for two weeks (without dosage information) [133]. In another case, after consuming Danshen for three days (without dosage information), a 66-year-old man who had been receiving warfarin 2–2.5 mg/day for nearly a year was hospitalized for bleeding accompanied with INR increasing from 2 to 5.5 [134]. Most mechanistic studies focus on the expression and metabolic activities of various cytochrome P450 enzymes. After rapidly and completely absorbed in GI tract, warfarin is metabolized mainly in the liver by CYP2C9, CYP1A2, and CYP3A4 in human. Clinical studies have been conducted to investigate the effects of Danshen or its single component on the metabolic activity of several CYP isoenzymes. A sequential, open-label, two-period clinical investigation indicated that Danshen may have induction effect on CYP3A and CYP1A2 [135]. Danshen or its components could also alter the distribution of warfarin. After entering into the blood, around 99% warfarin would bind to the plasma protein, mainly albumin, to form a warfarin-albumin complex that has no therapeutic effect. Danshen and its major component danshinone IIA could competitively bind to albumin and therefore inhibit the protein binding of warfarin [136]. Reduced protein binding may result in over-anticoagulant because of the increased blood concentration of warfarin. Though less investigated, the pharmacodynamics effects of Danshen on the warfarin cannot be ignored. Danshen is widely used for removing blood stasis to improve the blood flow. With the similar clinically therapeutic effect to warfarin, concomitant use of warfarin and Danshen may cause a synergistic effect and result in over-anticoagulation. On basis of previous studies, interaction between Danshen and warfarin can be considered as* highly probable*. Therefore, it may be prudent to advice against concurrent use of Danshen with warfarin.

#### 3.2.3. Lycium (*Lycium barbarum*)

Lycium have anti-inflammatory, antioxidation, and anticancer properties. It could be used for diabetes, hypertension and erectile dysfunction [137].* In vitro* study found that Lycium was a weak CYP2C9 inhibitor, but it was insufficient to cause a drug interaction [137]. A Chinese women stabilized on warfarin had a significantly rise in her INR from 2 to 4.1 when concurrently taking Lycium (3-4 glasses daily) for 4 days. And the INR returned to normal when herbal treatment stopped [138]. In another case, after 4-day coadministration of Lycium juice (30 mL each morning and evening) and warfarin, a 71-year-old woman had nosebleeds, bruising, and rectal bleeding [139]. These cases reports showed that Lycium might potentiate effects of warfarin. Warfarin is mostly metabolized by CYP2C9. Inhibition of CYP2C9 may therefore lead to increased warfarin levels and effects. However, other mechanisms cannot be ruled out. On basis of the INR changes in several cases, the interaction between warfarin and Lycium may be considered as* possible*.

In addition, herbs preclinically evidenced to affect pharmacokinetics of warfarin (Andrographis [140–143],* Horse chestnut* [63, 82],* Schisandra* [144–146],* Gegen* [147–149], and* Liquorice* [150, 151]) and pharmacodynamics of warfarin (*Clove* [40, 152, 153],* Lapacho* [32, 154]), or both (*Evening primrose* [70, 155],* Feverfew* [60, 107, 156]); herbs containing vitamin K (Alfalfa [40, 157, 158],* Asparagus* [40, 159]) or coumarin (*Bogbean* [18, 40],* Celery* [160, 161], and* Horse chestnut* [40]) and herbs with similar or opposite pharmacological actions to those of warfarin may also interact with warfarin. As no clinical evidence is now available to support an interaction, these herbs were defined as* doubtful* in the current review.

## 4. Discussions

The basic issues involved in assessing the importance of interactions between herbs and drugs are similar to those in evaluating interactions between conventional drugs, but for herbal medicines the picture is complicated by their very nature: the herbs are complex mixtures and there is also lack of reliable information about the occurrence and relevance of interactions. This review attempts to answer following questions.

- Is the herb-warfarin interaction clinically evidenced or only theoretical and speculative?
- If they do interact, how serious is it?
- Has this interaction been identified many times or only once?
- What is the possible mechanism for the interaction?

The current review scales the clinical severity and evidence reliabilities of herb-warfarin interaction according to previously validated criteria. The uniqueness of this study is demonstrated in Table 3, which summarizes clinical effects of proposed herb-warfarin interaction, severity, and possibility of these interactions as well as their possible mechanisms. The outcome can be harmful if the interaction causes an increase in the effect of warfarin. A potential example of this is bleeding related to coadministration of cranberry with warfarin. However, a reduction in warfarin efficacy due to an interaction can sometimes be just as harmful as an increase. For example, the reduction in warfarin effect caused by St. John's wort may lead to thrombosis. In regard to this, it seems extremely dangerous for patients to take warfarin and herbs together. But this could be an overestimation of the outcome. First, human beings do not respond uniformly to drugs or herbal medicines due to many elements including genetic makeup, sex, age, diseases, renal and hepatic functions, ethnic background, nutritional state, and other factors. Second, a good deal of evidence on herb-warfarin interactions discussed in this paper is based on case reports, which are sometimes incomplete and do not allow one to infer a causal relationship. According to our current review, out of 38 herbs with clinical evidence, only 4 interactions are regarded as* highly probable*. It is worth noting that even documented case reports could never establish a causal relationship between herb administration and an adverse event, as sometimes only one case report has been used, and in many cases, the quality of documented case report is poor. The often underregulated quality of herbal medicines is another safety issue. Contamination or adulteration of herbal medicines, including adulteration with synthetic drugs, may be relatively frequent and can cause drug interactions. In other words, the possibility that a contaminant adulterant instead of herbal ingredient causing drug interactions cannot be ruled out. Therefore, it is difficult to make conclusion on whether or not coadministration of certain herb with warfarin contributes to the adverse event. Although it is impossible to identify all clinically important herb-warfarin interaction, some general principles can be reached as follow.

- Herbs containing large amount of vitamin K have high possibility to interact with warfarin.
- Blood vitalizing herbs and herbs with antiplatelet effects are liable to have interaction with warfarin.
- The elderly are at greater risk because of reduced liver and renal function on which drug clearance depends.

Since herb usage could be quite variable, the current review only focuses on single herb without herb remedies included. In addition, variability in the dosage of both warfarin and proposed herbs is critical for managing an interaction. As a narrow therapeutic drug, the dosage of warfarin is adjusted according to the INR of patient. For therapeutic purpose, the value of INR should be maintained at a range of 2 to 3. While for healthy subjects, the normal value of INR is 0.9 to 1.2. For most of the clinical studies mentioned in current review, warfarin doses and intensity of anticoagulation were stable before initiation of herbs. Therefore the intensity of interactions was mainly correlated with the dosage of coadministrated herbs. Unfortunately, most of the existing case reports failed to mention the relevant dosage of interacting herbs. For example, a report describes an intracerebral hemorrhage, which occurred in an elderly woman within 2 months of her starting to take ginkgo. She had been taking warfarin uneventfully for 5 years. The author of the report speculated that ginkgo may have contributed towards the hemorrhage [83]. However, the dosage of ginkgo was not reported. Conversely, in a randomized, crossover study in 21 patients stabilized on warfarin, a tablet of* Ginkgo biloba* 100 mg daily for 4 weeks, did not alter the INR or the required dose of warfarin, when compared with placebo [81]. Therefore, despite the prevalence report about herb-warfarin interaction, the intensity of these interactions may be overestimated. All the herbal medicines have side effects more or less, but when used at a therapeutic dosage in clinical treatment, it can be accepted as a safe medicine. On this basis, the authors suggest further studies with corresponding information on herb dosage. In addition, herbs of different producing regions, medical parts, and processing techniques may contain various amounts of active compounds. Therefore, it is reasonable to develop qualified and standardized herbal products such as EGb 761, a standardized and commercially available extract of* Ginkgo biloba* leaves.

## 5. Conclusions

An overview of the clinical data regarding herb-warfarin interactions was conducted in this paper, highlighting clinical effects, severity of documented interaction, and quality of clinical evidence. Among thirty-eight of selected herbs, four were evaluated as* highly probable* to interact with warfarin (Level I evidence), three were* probable* interaction (Level II evidence), and ten and twenty-one were* possible* (Level III evidence) and* doubtful* (Level IV evidence), respectively. Herbs defined as* highly probable* (Cranberry, Soya, St John's wort, and Danshen) and* probable* (coenzyme Q10, Chinese Angelica, Ginger) are strongly suggested to be avoided from concomitant use with warfarin. For herbs defined as* possible and doubtful*, although insufficient evidences supporting the interaction yet, for safety reason, it is recommended to closely monitor INR in patients taking warfarin. Patients and physicians are advised to use herbal medicines within a safety dosage. Qualified and standardized herbal products such as EGb 761 are recommended for scientific researches, especially for clinical studies. Although several corresponding pharmacokinetic or pharmacodynamic mechanisms of interactions were able to be identified for a small amount of “interacting herbs,” there are still a great number of unexplored aspects of herb-warfarin interactions. The clinical effects of herbs on warfarin therapy should be further investigated through multicenter studies with large sample sizes.

## Figures and Tables

**Table 1:** Scoring of clinical severity of herb-warfarin interaction [14].

Clinical severity	Potentiation	Inhibition
Major	Death, major bleeding, entailing entire cease of warfarin therapy	Occurrence of thrombosis

Moderate	INR increase, entailing adjustment in warfarin dosage INR increase to greater than 5.0 INR increase by greater than 1.5 units	INR decrease, entailing adjustment in warfarin dosage; INR decrease to less than 1.5 INR decrease by greater than 1.5 units

Minor	INR increase but requiring no change in warfarin dosage INR increase to no more than 5.0 INR increase by smaller than 1.5 units	INR decrease but requiring no change in warfarin dosage INR decrease to no more than 1.5 INR decrease by smaller than 1.5 units

Nonclinical	No change in INR	No change in INR

**Table 2:** Criteria for defining evidence reliabilities of an interaction [14, 16].

Reliabilities of evidence	Criteria required
I: highly probable	A, B, and C plus any one or more of D to G
II: probable	A, B plus one or more of C to G
III: possible	A plus one or more of B to G
IV: doubtful	Any combination of B to G or A alone

(A) Was the timing correct for an interaction to be pharmacologically plausible?	
(B) Did laboratory test (INR, prothrombin time, and thrombotest) support the contention of an interaction?	
(C) Were other potential factors affecting warfarin pharmacokinetic or pharmacodynamics ruled out?	
(D) Was there other objective evidence?	
(E) Was a dose-response relationship shown for the interacting herb?	
(F) Was the subject rechallenged and, if so, did a similar response occur?	
(G) Did the same thing happen on previous exposure to the herb?	

*Notes*:

A: in patient-based studies, warfarin must been taken at a stabilized dose before initiation of the interacting herbs. In addition, the potentially interacting herbs should be consumed long enough in usual doses to attain a substantial plasma level. For volunteer-based studies, subjects had to receive warfarin, both alone and with the interacting herbs, for similar periods.

B: in patient-based articles, the coagulation variable should be out of therapeutic range, whereas for volunteer studies, a change of at least 20% was required in coagulation parameters. For conclusion of “no interaction,” the absence of a statistically significant change in coagulation variables was required.

C: factors such as diet, other medications, or certain medical conditions, especially liver diseases, should be declared to be ruled out as possible causes of the outcome.

D: other objective evidences refer to changes in plasma level of warfarin or level of vitamin K dependent clotting factors (II, VII, IX, or X).

E: the alterations in the dose of the coadministrated interacting herbs correlated with subsequent changes in warfarin coagulation variables, inferring a dose-response relationship.

F: the interacting herb should be administered simultaneously with warfarin in two or more separate courses, with similar results for each course.

G: similar outcome should be found for the patient cousing the interacting herb with warfarin at a time prior to that reported.

**Table 3:** Summary of herb-warfarin interactions supported by clinical evidence.

Herbs (common and Latin name)	Clinical effects	Severity	Reliabilities of evidence	Mechanisms	
				PK	PD
Cranberry *(Vaccinium macrocarpon) *	Potentiation	Major [55]	I	F [52, 53, 160]	D [40]
Soya *(Glycine max *Merr.)	Inhibition	Moderate [115, 116]	I	F [113, 114]	B [115]
St John's wort *(Hypericum perforatum) *	Inhibition	Major [121, 122]	I	F [41, 118, 119]	NA
Danshen *(Salvia miltiorrhiza) *	Inhibition	Moderate [133, 134]	I	F, G [131, 132, 135, 136]	A,C [40]

Coenzyme Q10 *(Theobroma cacao) *	Inhibition	Minor [47, 48]	II	NA	B [50]
Chinese angelica *(Angelica sinensis) *	Potentiation	Moderate [126]	II	F [125]	C [40]
Ginger *(Zingiber officinale *Roscoe)	Potentiation	Moderate [161]	II	NA	A [162]

Chamomile *(Matricaria recutita) *	Potentiation	Major [44]	III	F [41, 163, 164]	NA
Chitosan *(Swertia chirayita) *	Potentiation	Moderate [46]	III	NA	B [45]
Cannabis *(Cannabis sativa *L)	Potentiation	Major [165]	III	F [166]	NA
Devil's claw *(Harpagophytum procumbens) *	Potentiation	Moderate [61]	III	F [60]	NA
Ginkgo *(Ginkgo biloba) *	Potentiation	Major [83]	III	F [75–77]	NA
Garlic *(Allium sativum) *	Potentiation	Major [40]	III	F [167, 168]	A [73, 169]
Ginseng *(Panax quinquefolius/Panax ginseng) *	Inhibition	Moderate [170]	III	F [40]	A [87]
Grapefruit *(Citrus paradise) *	Potentiation	Major [94]	III	F [95]	NA
Green tea *(Camellia sinensis) *	Inhibition	Moderate [97]	III	NA	B [99]
Lycium *(Lycium barbarum) *	Potentiation	Major [139]	III	F [137]	NA

Boldo *(Peumus boldus) *	Potentiation	Minor [36]	IV	NA	C [40]
Echinacea *(Echinacea purpurea) *	Inhibition	Minor [66]	IV	F [119, 171]	NA
Fenugreek *(Trigonella foenum-graecum) *	Potentiation	Minor [36]	IV	NA	B, C [40]
Melilot *(Melilotus officinalis) *	Potentiation	Moderate [102]	IV	NA	C [40]
Parsley *(Petroselinum crispum) *	Potentiation	Moderate [104]	IV	F [103]	B [40]
Pumpkin *(Cucurbita pepo) *	Potentiation	Minor [106]	IV	NA	B [40]
Red clover *(Trifolium pretense) *	Potentiation	Major [108]	IV	F [60, 107]	NA
Saw palmetto *(Serenoa repens) *	Potentiation	Minor [106, 111]	IV	F [109]	NA

*Notes*. (1) As to mechanisms of herb-warfarin interaction, PD factors including the following: A: interference with platelet function; B: altering gut vitamin K synthesis or containing vitamin K; C: interference with vitamin K cycle; D: interference with coagulation cascade. PK factors including the following: E: interference with warfarin absorption; F: interference with metabolizing enzymes of warfarin; G: interference with protein binding of warfarin. (2) Other nonclinical evidenced herbs defined as doubtful in Section 3 were excluded in this table.
